# Quantification of resistant alleles in the β-tubulin gene of field strains of gastrointestinal nematodes and their relation with the faecal egg count reduction test

**DOI:** 10.1186/s12917-017-0992-9

**Published:** 2017-03-20

**Authors:** Myriam Esteban-Ballesteros, Francisco A. Rojo-Vázquez, Philip J. Skuce, Lynsey Melville, Camino González-Lanza, María Martínez-Valladares

**Affiliations:** 1 Departamento de Sanidad Animal, Facultad de Veterinaria, Universidad de León, Campus de Vegazana s/n, 24071 León, Spain; 2 Instituto de Ganadería de Montaña (CSIC-Universidad de León), Finca Marzanas, Grulleros, 24346 León, Spain; 3 Moredun Research Institute, Pentland Science Park, Bush Loan, Edinburgh, UK

**Keywords:** Sheep, *Teladorsagia circumcincta*, *Trichostrongylus colubriformis*, Anthelmintic resistance, FECRT, Single nucleotide polymorphism, Beta-tubulin, Pyrosequencing

## Abstract

**Background:**

Benzimidazole (BZ) resistance in gastrointestinal nematodes is associated with a single nucleotide polymorphism (SNP) at codons 167, 198 and 200 in the isotype 1 of beta-tubulin gene although in some species these SNPs have also been associated with resistance to macrocyclic lactones. In the present study we compared the levels of resistance in *Teladorsagia circumcincta* and *Trichostrongylus colubriformis* by means of the faecal egg reduction test (FECRT) and the percentage of resistant alleles obtained after pyrosequencing. The study was conducted in 10 naturally infected sheep flocks. Each flock was divided into three groups: i) group treated with albendazole (ABZ); ii) group treated with ivermectin (IVM); iii) untreated group. The number of eggs excreted per gram of faeces was estimated at day 0 and 14 post-treatment.

**Results:**

Resistance to ABZ was observed in 12.5% (1/8) of the flocks and to IVM in 44.4% (4/9) of them. One flock was resistant to both drugs according to FECRT. Coprocultures were performed at the same dates to collect L3 for DNA extraction from pooled larvae and to determine the resistant allele frequencies by pyrosequencing analysis. In *T. circumcincta*, SNPs were not found at any of the three codons before treatment; after the administration of ABZ, SNPs were present only in two different flocks, one of them with a frequency of 23.8% at SNP 167, and the other 13.2% % at SNP 198. In relation to *T. colubriformis*, we found the SNP200 before treatment in 33.3% (3/9) of the flocks with values between 48.5 and 87.8%. After treatment with ABZ and IVM, the prevalence of this SNP increased to 75 and 100% of the flocks, with a mean frequency of 95.1% and 82.6%, respectively.

**Conclusion:**

The frequencies observed for SNP200 in *T. colubriformis* indicate that the presence of resistance is more common than revealed by the FECRT.

## Background

Infections by gastrointestinal nematodes (GIN) are a serious problem for extensive systems of sheep farming worldwide. GIN can cause serious losses in animal production since they affect the production of milk, wool and meat, and also interfere with reproduction [1]. The most prevalent GIN species infecting sheep in temperate areas of the world are *Teladorsagia circumcincta*, *Trichostrongylus* spp., *Haemonchus contortus, Chabertia ovina* and *Cooperia* spp.

The usual mode of controlling GIN infections in ruminants is by chemotherapy and the most commonly used anthelmintics are grouped into 3 families: benzimidazoles (BZs), imidazothiazoles and macrocyclic lactones (MLs). Recently, two new chemical groups have been introduced to the market, namely, the aminoacetonitrile derivative (monepantel) [2] and the spiroindole (derquantel), in combination with abamectin [3]. Anthelmintics have been used with great success in the past. However, their frequent use and the underdosing of animals has favored the emergence of anthelmintic resistance (AR), among other factors. There are some reports describing AR against all anthelmintic groups worldwide, even against the most recent anthelmintic drug, monepantel, in New Zealand, The Netherlands and Uruguay in *T. circumcincta*, *Trichostrongylus colubriformis* and *H. contortus* [4–7]. Therefore, the high prevalence of AR to several drugs against GIN in small ruminants continues to threaten the viability of small ruminant farms [8].

The BZs and MLs are the major groups of anthelmintics used to control GIN infections in some countries like Spain [9]. The mode of action of BZ involves binding to β-tubulin and disrupting microtubule polymerization of tubulin [10–13]. Genetic studies have shown that BZ resistance is associated with a single nucleotide polymorphism (SNP) in the gene encoding isotype-1 β-tubulin. The substitution of a phenylalanine (Phe, TTC) for a tyrosine (Tyr, TAC) at codon 200 (F200Y) has been linked to BZ resistance in *H. contortus*, *T. colubriformis, T. circumcincta, C. oncophora* and *O. ostertagi* [14]. Less frequently, the same SNP was found at codon 167 (F167Y) in resistant strains of *H. contortus*, *T circumcincta* and *O. ostertagi* [15]. Furthermore, a point mutation of alanine (Ala, GCA) to glutamine (Glu, GAA) at codon 198 has been described in resistant strains of *H. contortus*, *C. oncophora* and *O. ostertagi* [16].

Previous assays have shown that resistant strains of *H. contortus* also carried the resistant allele at codons 200 and 167 after treatment with an ML, specifically, ivermectin (IVM). These results suggest a possible association between resistance mechanisms in ML and BZ [17].

Since AR is increasing recently around the world [8, 18, 19], its control is required with the aim to use anthelmintic drugs more effectively and sustainably and to avoid the development of new resistant strains. The faecal egg count reduction test (FECRT) is the most widely used diagnostic method for the detection of AR in vivo [20, 21]. However, this technique lacks sensitivity and is not able to detect resistance when the level of genetically resistant individuals in the population is below ~25% [22]. Therefore, the development of new in vitro techniques is necessary for an early diagnosis of AR.

In this context, the aim of the present study was the measurement of the frequency of the resistant allele at codons 200, 198 and 167 of isotype-1 β-tubulin in GIN field isolates of sheep flocks from the Northwest of Spain before and after administration of a BZ and/or ML anthelmintic.

## Methods

### Faecal egg count reduction test (FECRT)

The study was conducted on 10 sheep flocks located in the province of León, Northwest Spain. In each flock, two groups of 10 sheep, naturally infected by GIN, were selected. Each group was treated with a BZ, albendazole (ABZ) (7.5 mg/kg bw), or IV (0.2 mg/kg bw). Faecal samples were collected on day 0 and day 14 post-treatment (pt). The number of eggs per gram of faeces (EPG) was determined by the modified McMaster method [23]. The faecal egg count reduction was calculated according to the recommendations of WAAVP (World Association for the Advancement of Veterinary Parasitology) [24] and using the following formula:

$$ \mathrm{FECRT}\ \% = \left(\mathrm{Arithmetic}\ \mathrm{mean}\ \mathrm{ep}\mathrm{g}\ \mathrm{day}\ 0 - \mathrm{Arithmetic}\ \mathrm{mean}\ \mathrm{ep}\mathrm{g}\ \mathrm{day}\mathrm{s} + 10\hbox{--} 14\right)/\mathrm{Arithmetic}\ \mathrm{mean}\ \mathrm{e}\mathrm{p}\mathrm{g}\ \mathrm{day}\ 0 \times 100 $$

When the percentage reduction in egg count was <90%, the flock was considered resistant to the anthelmintic; if the faecal reduction was between 90 and 95%, the flock was classified as borderline or suspicious of resistance, and when values were higher than 95%, the flock was considered susceptible to the anthelmintic.

From each group, pooled faeces were cultured on days 0 and 14 pt to recover third stage larvae (L3). After collecting the cultures, a minimum of 100 L3 per culture were identified using the morphological keys in MAFF [23]; in flocks with a reduction of 100% we recovered a few L3, between 20 and 225. The remainder was stored at −20 °C until the DNA extraction was carried out.

### DNA extraction

DNA was extracted from each L3 pool, before and after treatment, using the Speed Tools Tissue DNA Extraction kit (Biotools), according to the manufacturer’s instructions. The DNA samples were stored at −20 °C until used.

### Determination of allele frequencies

Two pairs of PCR primers were designed to amplify two fragments of the gene encoding the β-tubulin of *T. circumcincta* including the SNPs 167 and 198/200, respectively (Table 1). For *Trichostrongylus colubriformis*, we only designed one pair of primers to amplify a fragment encompassing codons 198 and 200 jointly (Table 1).

**Table 1:** Primer sequences for *T. circumcincta* SNPs 167 and 198/200, and *T. colubriformis* SNP 198/200, and sequence primers for both species

Primer name	Sequence 5′-3′	Modifications	Expected product size (pb)	Tm (°C)
*T. circumcincta*				
Tc SNP200 F	CACTCTTTCTGTACACCAATTG	[Btn]5	128	60
Tc SNP200 R	AGTGATTGAGATCGCCATAA	-		
Tc SNP167 F	CAAAATTCGCGAGGAGTAT	-	276	60
Tc SNP167 R	TTCTACCAATTGGTGTACAGAAAG	[Btn]5		
*T. colubriformis*				
Tri SNP200 F	TACTTTATCAGTCCATGAGCTGG	[Btn]5	128	62
Tri SNP200 R	ATGGTTGAGATCTCCATAGGTTG	-		
*Sequencing*				
Tc 200 Seq	AGAGCTTCATTATCGATG	-	-	60
Tc 167 Seq	CGGATAGAATCATGGCT	-	-	60
Tri 200 Seq	AGAGCTTCGTTATCGATGCA	-	-	62

With the aim to amplify these three fragments, we firstly carried out a PCR with the primers described in Table 1, but without biotinylation. Cycling conditions were 95 °C for 10 min followed by 40 cycles of 95 °C for 30 s, Tm for 30 s and 72 °C for 45 s followed by 10 min at 72 °C and 4 °C to finish using *Taq* DNA polymerase MasterMix 2x, MgCl_2_ 2,0 mM (Biotools, Madrid, Spain). PCR products were run on a 1.5% agarose gel, and the corresponding band was cut out and purified using PCR Clean-Up SpeedTools kit (Biotools, Madrid, Spain). The final elution volume was 50 μl. Then, this same PCR was carried out again in a 50 μl reaction volume but, in this case, one of the primers of each pair was labeled with biotin at the 5´, as shown in Table 1. Prior to pyrosequencing, a 7 μl aliquot of each PCR product was tested by agarose gel electrophoresis.

The specificity of the primers used to amplify these regions was confirmed initially after the amplification of the two regions in *T. circumcincta* adult worms and of one region in *T. colubriformis* adult worms. These PCRs were run in the same way as previously described but using 1 μl of DNA template. The resulting bands were excised from an agarose gel 1,5% and purified using PCR Clean-Up SpeedTools kit (Biotools) and then were sequenced in the “Laboratorio de Técnicas Instrumentales” (University of León, Spain).

Pyrosequencing of the three resulting PCR fragments, targeting SNPs 167 and 198/200 in *T. circumcincta* and SNPs 198/200 in *T. colubriformis*, was carried out using the sequencing primers (Seq) (Table 1). The sequencing primer for the SNP167 of *T. circumcincta* was previously described by Skuce et al. [25]. The pyrosequencing assay was carried out using a PyroMark ID Pyrosequencer (Biotage, Sweden) according to the manufacturer’s recommendations. After the initial PCR amplification, 40 μl of PCR product was added to 37 μl 2x Binding buffer (Biotage, Sweden), 3 μl streptavidin sepharose beads (Roche) in a 96 well plate and then agitated for 5 min at room temperature to allow binding of biotin-labelled DNA to the beads. The beads were processed using the sample preparation tool and reagents (Biotage, Sweden) dispensed into the assay plate with 40 μl of 0.4 μM sequencing primer per well. Positive controls representing gDNA extracted from susceptible and resistant individual adults from each species were included in each assay.

The determination of the allele frequencies was carried out at least 3 times for each sample and the arithmetic mean was calculated. The frequencies of the resistant allele with values equal to or lower than <5% were considered as technical background and, therefore, not classed as resistant.

## Results

### Faecal egg count reduction test (FECRT)

The results of the FECRT carried out in the 10 sheep flocks are shown in Tables 3 and 4.

According to the results of FECRT, and taking into account the resistant and borderline flocks together, 40% of flocks were resistant to one drug. Resistance to ABZ was observed in 12.5% (1/8) of the flocks and to IVM in 44.4% (4/9). One flock (10%) was resistant to both drugs.

Before treatment, the most frequent species were *T. circumcincta* (43–55% of larvae) and *Trichostrongylus* sp (38–47% of larvae), however, other GIN species such as *H. contortus*, *Bunostomun* sp, *Nematodirus* sp, *Chabertia ovina* and *Cooperia oncophora* were also observed at a lower percentage (1-13% of larvae) (Table 2). After treatment, only *T. circumcincta* and *Trichostrongylus* sp were identified, with the exception of farm 7 where *Bunostomun* sp was also identified (6% of larvae) (Tables 3 and 4).

**Table 2:** Allele frequencies before treatment for *T. circumcincta* SNPs 167 and 198/200, and *T. colubriformis* SNP 198/200, and morphological identification

Farm	*T. colubriformis*		*T. circumcincta*			% L3 species						
	% SNP200	% SNP198	% SNP200	% SNP198	% SNP167	Tc	Tri	Hc	Bu	Ne	Ch	Co
1	0	2.5	0	0	0	51	42	4	1	2	0	0
2	51.6	6.8	0	0	-	49	47	3	0	1	0	0
3	-	-	3.4	0	0	52	45	2	0	0	0	1
4	0	3.3	0	0	2.6	43	41	2	12	1	2	0
5	0	0	0	0	2.8	55	42	2	0	0	0	0
6	3.1	0	0	0	2.8	48	38	0	1	0	13	0
7	2	2	0	2	3.1	46	43	0	3	0	8	0
8	87.8	3.9	-	-	0	-	-	-	-	-	-	-
9	0	3.1	0	0	-	54	46	0	0	0	0	0
10	48.5	0	0	0	-	53	46	0	1	0	0	0

Tc: *T. circumcincta,* Tri: *Trichostrongylus* spp, Hc: *H. contortus*, Bu: *Bunostomun* spp, Ne: *Nematodirus* spp, Ch: *Chabertia ovina* and Co: *Cooperia oncophora*. -: Failed samples

**Table 3:** Allele frequencies after ABZ treatment for *T. circumcincta* SNPs 167 and 198/200, and *T. colubriformis* SNP 198/200, and morphological identification

Farm	% Egg reduction	R/S classification	*T. colubriformis*		*T. circumcincta*			% L3 species		
			% SNP200	% SNP198	% SNP200	% SNP198	% SNP167	Tc	Tri	Bu
1	91.4	Borderline	1.7	4.8	2.9	0	3	50	50	0
2	98.9	S	97.6	3	0	0	23.8	55	45	0
3	100	S	96	1.5	0	13.2	4.4	56	44	0
4	98.5	S	99.3	5.5	0	0	-	58	42	0
5	100	S	89.8	0	0	0	4.8	52	48	0
6	99.3	S	1.3	3	-	-	-	62	38	0
7	100	S	90.6	19	-	-	0	47	47	6
10	100	S	97.2	4.8	-	-	0	38	62	0

Tc: *T. circumcincta,* Tri: *Trichostrongylus* spp and Bu: *Bunostomun* spp. -: Failed samples

**Table 4:** Allele frequencies after IVM treatment for *T. circumcincta* SNPs 167 and 198/200, and *T. colubriformis* SNP 198/200, and morphological identification

Farm	% Egg reduction	R/S classification	*T. colubriformis*		*T. circumcincta*			% L3 species	
			% SNP200	% SNP198	% SNP200	% SNP198	% SNP167	Tc	Tri
1	37.3	R	23.3	6.4	0	0	0	63	37
2	100	S	97.3	0	0	0	0	54	46
3	86.0	R	79.2	14	0	0	2.4	41	59
4	−11.9	R	94.7	0	0	0	0	60	40
5	99.3	S	93.7	1	-	-	0	50	50
6	100	S	-	-	0	0	3	54	46
7	95.9	S	100	14	0	0	0	70	30
8	100	S	89.9	6.1	-	-	0	-	-
9	93.5	Borderline	95	2.4	0	0	6.1	44	56

Tc: *T. circumcincta* and Tri: *Trichostrongylus* spp. -: Failed samples

### β-Tubulin allele frequencies

The assays were capable of detecting BZ resistance-associated SNPs because, with gDNA from resistant adult samples, included as a positive control, the mean frequencies of the resistant allele at codon 200 were 89% in *T. circumcincta* and 92% in *T. colubriformis*.

#### Allele frequencies before treatment

In relation to *T. circumcincta,* the resistant allele was not found in any of the flocks tested at codons 167, 198 or 200. In *T. colubriformis*, the resistant allele was found in 11.1% (1/9) of the flocks at codon 198, with a very low percentage (6.8%). At codon 200, the resistant allele was found in 33.3% (3/9) of the flocks, with high frequencies ranging from 48.5 to 87.8% (Table 2).

#### Allele frequencies after treatment with ABZ

For *T. circumcincta*, after the administration of ABZ, the percentage of flocks with the resistant allele at codon 167 was 16.7% (1/6), with a frequency of 23.8%. The resistant allele at codon 198 was found in one out of five flocks (20%) with a frequency of 13.2%. On the other hand, the resistant allele carrying SNP200 was not found in any flock.

In relation to *T. colubriformis*, the resistant allele at codon 198 was found in 25% (2/8) of flocks, with values of 5.5 and 19%, and at position 200 in 75% (6/8) of flocks with very high frequencies, between 89.8 and 99.3% (Table 3).

#### Allele frequencies after treatment with IVM

After treatment with IVM, for *T. circumcincta*, the resistant allele carrying SNP167 was found in 11.1% of the flocks (1/9) but with a very low frequency (6.1%). At codons 198 and 200, the resistant allele was not found in any sample.

For *T. colubriformis*, the SNP198 was found in 3 of the 8 flocks (37.5%) at low frequency, between 6.1 and 14%. At codon 200, all flocks in which the determination was done carried the resistant allele, with frequencies ranging from 23.3 to 100% (Table 4).

## Discussion

This study describes the frequencies of the resistant alleles present in the gene encoding isotype-1 β-tubulin of field populations of GIN, collected before and after treatment with ABZ or IVM. The resistance status of these flocks situated in the Northwest of Spain was firstly determined in vivo by means of the FECRT.

Since the FECRT cannot detect low resistance levels, especially under field conditions when the infections by GIN are typically of mixed species composition [26], determining the frequency of resistant alleles in pools of L3 could be an alternative to or proxy for the FECRT, with the added benefit, if sufficiently robust and repeatable, of not needing to treat animals to determine resistance status. Since the resistant phenotype is only detected by the FECRT when the frequency of resistant alleles in the population is over 25% [22], the FECRT only represents an estimate of the resistance in a flock naturally infected by GIN.

In this study, we used pyrosequencing to determine the resistant allele frequency in the gene encoding isotype-1 β-tubulin at codons 167, 198 and 200 of *T. circumcincta* and codons 198 and 200 of *T. colubriformis*, in DNA samples from pools of L3 collected before and after treatment. These species were the most frequent detected in all sampled flocks and represent the 84–100% of the GIN burden on all of them.

According to Kwa et al. [14] and Elard and Humbert [27], the mutation of Phe to Tyr at position 200 of β-tubulin isotype 1 is the most important mechanism responsible for conferring resistance to BZ. According to Silvestre and Cabaret [15], this same mutation at codon 167 is also involved in the development of resistance in the absence of the mutation at position 200. The SNP167 is rare in field populations, but for *T. circumcincta* may account for the survival of parasites which do not carry the mutation at codon 200 [15]. In the present study, this SNP was present before treatment; after treatment with ABZ, it was only shown in one susceptible flock with a low frequency of 23.8% and, therefore, we do not correlate it with AR. However, Demeler et al. [28] found resistant alleles at codons 167 and 200, but not at 198 in an *Ostertagia ostertagi* BZ resistant isolate, indicating that BZ treatment does not necessarily select for the SNP198. In the present study, the SNP at codon 198 was not found before treatment in *T. circumcincta* and, after administration of ABZ, only one susceptible flock, with a low frequency (13.2%) was detected in the five flocks tested. Our results regarding the SNP198 in *T. circumcincta* are not conclusive, due to the low number of analyses. However, Ghisi et al. [16] found the SNP198 in 90% of resistant isolates of *H. contortus* which did not carry the mutation in codon 200. Therefore, SNP198 has been related to BZ resistance. In *T. colubriformis*, before treatment, SNP198 was only found in one flock with a very low frequency (6.8%), and after treatment with ABZ in two different flocks with frequencies of 5.5 and 19%.

Therefore, in the present study, the frequencies of resistant alleles carrying SNPs 167 and 198 in *T. circumcincta*, and SNP 198 in *T. colubriformis*, are either zero or very low and consequently we did not find any relation between them and AR status/phenotype.

In relation to the SNP200 in *T. circumcincta*, this was not found before or after treatment with ABZ. These results are in agreement with the previous study of Martinez-Valladares et al. [29], who did not describe any resistant allele at codons 167, 198 and 200 in individual *T. circumcincta* L3 collected from flocks in the same study area (León, Spain). Due to the absence of resistant alleles, but also because we only found one flock with borderline resistance to ABZ, we cannot conclude that there is an association between resistance and resistant allele frequency at codon 200 in *T. circumcincta*. On the other hand, Skuce et al. [25], after studying different strains of *T. circumcincta*, reported a resistant allele frequency of 64.9% in a multidrug-resistant strain (MTci5) at codon 200 and 0% in a susceptible strain (MTci1).

However, the results shown in the current study for the frequencies at SNP 200 in *T. colubriformis* are totally different. Before treatment with ABZ, the resistant allele was present in 33.3% of the farms tested, with values between 48.5 and 87.8% (Mean = 62.6%) and, after ABZ treatment, in 75% of the farms, with very high allele frequencies, ranging from 89.8 to 99.3% (Mean = 95.1%). However, these data are not consistent with the results of the FECRT, since we found high levels of the resistant allele in susceptible farms classified according to the FECRT. This result could be due to the resistant allele(s) being diluted in the population because of the presence of other (susceptible) species, before treatment. The FECRT can only detect resistance when ~25% of the total population is resistant. The resistant allele at codon 200 has been previously reported in different BZ resistant strains of *T. colubriformis* [30, 31] and also in *Trichostrongylus axei* adult worms, with a frequency of 63% recovered from lambs after treatment with BZ [32].

In the current study, we also determined the frequency of the resistant alleles for these species before and after the administration of IVM. Freeman et al. [33] were the first authors to describe a relation between resistance to IVM and the β-tubulin gene in *H. contortus*. These authors found a marked alteration in the amphid neurons, which are formed by bundles of microtubules, heterodimers of α-tubulin and β-tubulin in IVM resistant strains of *H. contortus*. Moreover, Mottier and Prichard [17] reported that repetitive use of IVM and moxidectin in *H. contortus* strains produced changes in the frequency of the alleles at codons 167, 198 and 200 of β-tubulin.

After the administration of IVM, in *T. circumcincta* the resistant allele was not found at codons 198 and 200. At position 167, the resistant allele was found in only one flock but with a very low frequency of 6.1%. These results are in agreement with a previous study in which none of these SNPs (167, 198 and 200) were detected in *T. circumcincta* when sheep were treated with IVM; the authors concluded that other molecular mechanisms than beta-tubulin could be implicated in the development of resistance against IVM in this case [29]. The present study would add further evidence to support this hypothesis, since 4 out of 9 studied flocks were resistant or borderline to IVM in the absence of the acknowledged resistant alleles. Therefore, we suggest that *T. circumcincta* is not responsible for the presence of resistance in these flocks and/or there could be different mechanisms or genes implicated in resistance development, especially to IVM. Previous studies suggest that the F200Y mutation could be related to IVM resistance in different species as observed by Njue et al. [34] in IVM-resistant strains of *C. oncophora* and by Eng and Prichard [35] in *Onchocerca volvulus*.

In contrast, for *T. colubriformis*, the frequency of the resistant allele at codon 200 after the administration of IVM was between 23.3 and 100% (Mean = 82.6%). These data suggest that there could be an association between the SNP 200 in *T. colubriformis* and IVM resistance. However, according to FECRT, only 4 flocks were resistant or borderline resistant to this drug. The effect of the resistant allele could be diluted by the presence of other susceptible species, like it happened after the treatment with ABZ. Recently, Ashraf et al. [36] reported that that IVM binds to *H. contortus* α and β tubulins at low micromolar affinities and stabilizes the microtubules. However, in a different study, Ashraf et al. [37] concluded that the SNPs 167 and 200 cause no difference in the polymerization of wild and mutant tubulins, and therefore, neither of the SNPs reduced IVM binding. The hypothesis is that the SNPs 167 and 200 could be part of a signaling mechanism that results in over expression of P-glycoproteins, which ultimately leads to IVM resistance [37].

In relation to these results, we suggest that the presence of resistance is more common than the FECRT indicates. Therefore using only this in vivo test we could obtain false negatives. The percentage of resistant alleles show a more detailed perspective on the presence of resistance at the molecular level; in consequence the level of resistance of a strain will depend on the percentage of resistant alleles in each species. Therefore, it would be worthy monitoring the percentage of resistant alleles after the FECRT, in susceptible flocks to avoid the development of resistance to BZs, and in resistant ones to dilute the percentage of resistant alleles, in both cases applying different management practices at the same time.

## Conclusions

In conclusion, comparing the results of FECRT and pyrosequencing, we suggest that the presence of resistance is more common than expected and that would have been declared using FECRT alone. The SNP200 in *T. circumcincta* is not present in the Spanish nematode populations tested although its association with resistance has been described in others field isolates from other countries, most notably, UK [25] and France [31]. In contrast, in *T. colubriformis*, the SNP200 is present on multiple farms and at high frequency before (between 48.5 and 87.8%) and after treatment with ABZ (mean in positive flocks = 95.1%) and IVM (mean = 82.6%). In relation to the other SNPs, 167 and 198, these were not detected in most of the analyses, so were either not present or present at very low frequency i.e. below the sensitivity of detection of the pyrosequencing assays used. Therefore, we cannot conclude that these SNPs are related with ABZ and/or IVM resistance in any of the Spanish GIN populations tested.

## Abbreviations

ABZ: Albendazole
AR: Anthelmintic resistance
BZ: Benzimidazole
BZs: Benzimidazoles
EPG: Eggs per gram of faeces
FECRT: Faecal egg count reduction test
GIN: Gastrointestinal nematodes
IVM: Ivermectin
L3: Third stage larvae
MLs: Macrocyclic lactones
SNP: Single nucleotide polymorphism
WAAVP: World association for the advancement of veterinary parasitology
